# Physical Fitness in World-Class Shot Put Para Athletes During Six Months of Training: A Longitudinal Case Report

**DOI:** 10.3390/sports13090328

**Published:** 2025-09-15

**Authors:** Exal Garcia-Carrillo, Nikolaos Zaras, Lawrence W. Judge, Angeliki-Nikoletta Stasinaki, Esteban Aedo-Muñoz, Antonio Castillo-Paredes, Jairo Azócar-Gallardo, Rodrigo Yáñez-Sepúlveda, Rodrigo Ramirez-Campillo

**Affiliations:** 1 Department of Physical Activity Sciences, Universidad de Los Lagos, Osorno 5290000, Chile; 3 Department of Physical Education and Sport Science, School of Physical Education, Sport Science and Occupational Therapy, Democritus University of Thrace, 69100 Komotini, Greece; nzaras@phyed.duth.gr; 2 School of Education, Faculty of Human Sciences, Universidad Bernardo O’Higgins, Santiago 8370993, Chile; 4 School of Kinesiology, Ball State University, Muncie, IN 47306, USA; lwjudge@hotmail.com; 5 School of Physical Education and Sport Science, National and Kapodistrian University of Athens, 17237 Athens, Greece; agstasin@phed.uoa.gr; 6 Escuela de Ciencias de la Actividad Física, El Deporte y la Salud, Facultad De Ciencias Médicas, Universidad de Santiago De Chile, Santiago 8370003, Chile; esteban.aedo@usach.cl; 7 Grupo AFySE, Investigación en Actividad Física y Salud Escolar, Escuela de Pedagogía en Educación Física, Facultad de Educación, Universidad de Las Américas, Santiago 8370040, Chile; acastillop85@gmail.com; 8 Departamento Ciencias De La Actividad Física, Universidad De Los Lagos, Osorno 5290000, Chile; jairo.azocar@ulagos.cl; 9 Programa De Investigación en Deporte, Sociedad Y Buen Vivir (DSBv), Universidad De Los Lagos, Osorno 5290000, Chile; 10 Faculty of Education and Social Sciences, Universidad Andres Bello, Viña del Mar 2520000, Chile; rodrigo.yanez.s@unab.cl; 11 Faculty of Rehabilitation Sciences, Exercise and Rehabilitation Sciences Institute, Universidad Andres Bello, Santiago 7591538, Chile; rodrigo.ramirez@unab.cl; 12 Sport Sciences and Human Performance Laboratories, Instituto de Alta Investigación, Universidad De Tarapacá, Arica 1000000, Chile

**Keywords:** athletic performance, disabled persons, sports for persons with disabilities, adaptive sports, athletes, physical fitness, sports, exercise, human physical conditioning

## Abstract

The purpose of this longitudinal case report was to assess physical fitness changes in world-class shot put para athletes during six months of training. One male (age: 34.8 years; mass: 96.9 kg; height: 1.79 m; sport class: F42), and one female (age: 45.3 years; mass: 60.1 kg; height: 1.64 m: F54) shot put para-thrower were assessed during a 24-week periodized training program, including strength and power training, throws, and plyometrics. Monthly assessments included competitive shot put throwing performance, the medicine ball throw test, and upper-body maximal strength, while body composition was measured before and after the training period. Shot put throwing performance improved by 10.1% and 1.6% for the male and female athletes, respectively. Similarly, performance in the medicine ball throw test increased by 15.2% and 8.4% for the male and female athletes, respectively. Maximal strength increased by 10.3% (male) and 3.3% (female). Body composition changes included an increased lean mass (2.5%) and reduced sum of six skinfolds (−9.8%) in the male athlete, while the female athlete experienced decreased body mass (−2.5%) and skinfolds (−11.7%). World-class shot put para athletes can improve strength and power through a structured periodized training plan. Monitoring strength and performance indicators monthly effectively tracked training adaptations in elite para athletes.

## 1. Introduction

Designing training programs for para athletes involved in throwing sports is a multifactorial process including manipulation of several variables such as volume, intensity, rest intervals, and frequency, while the regulation of physical fitness development through resistance/weightlifting training, strength–power training, agility drills, and plyometrics, adds complexity to the overall planning process [1,2,3]. This complexity is amplified by the need to tailor strength–power development and periodization strategies to athletes’ specific impairments (e.g., seated vs. standing throwing) while optimizing training adaptations [4]. Although the theory of periodization may provide a solid solution to these physiological and methodological challenges [5], para-throwers face several everyday barriers that often hinder the smooth training process, mainly through difficulty in accessing sports facilities, discrimination, limited innovation, unaffordability, and unavailability of crucial assistive products [6,7]. These barriers not only limit training quantity but also quality: physiological adaptations are compromised when athletes cannot access impairment-specific equipment (e.g., throwing frames for class F54), while chronic stress from discrimination may elevate cortisol levels, impairing recovery and long-term performance [8]. These barriers and chronic stressors highlight the importance of both designing adaptive training programs and implementing monitoring strategies specifically tailored to para athletes.

Although there has been a significant amount of scientific research in evaluating the training load in high-performance sports in recent years [9], there is still not a gold standard monitoring tool that can be consistently considered definitive, accurate, and reliable [10]. In fact, the best approach to training monitoring may vary depending on the sport, and it is common to use multiple monitoring tools in an attempt to evaluate the training load and the training-induced adaptations required by athletes [11,12]. However, there is a lack of research data regarding the monitoring of training in para athletes, especially in para-throwers.

Para Athletics throwers spend a large part of their preparation in training programs including a variety of training modalities (i.e., specific throwing, resistance training, and strength–power training) [13,14,15] following the basic principles of periodization [16]. A recent study on para throwers showed that male para throwers usually train with a throwing frequency of 3 sessions per week, with a 120–130 min duration, and approximately 3–4 gym training sessions per week, totaling 6–7 h, while including medicine ball training sessions 1–3 times a week [17]. Additionally, similar findings were found for female para-throwers but with shorter throwing sessions (50–60 min) and less time dedicated to gym training (2–3 h) [17], similar to non-disabled throwers [3,18].

Monitoring these programs requires a combination of multiple tools to assess and track training adaptations in para throwers, similar to non-disabled throwers [19], while at the same time reducing the risk of injuries [20]. These external and internal training load metrics, such as the changes in bench press strength or the session’s rate of perceived exertion (sRPE), are critical for evaluating the mechanical demands placed on thrower athletes [19,21,22]. A primary goal of monitoring the training loads is to systematically inform the coach regarding the athlete’s readiness and make decisions about the training plan [23]. Additionally, variables of training monitoring can be important means of reducing the risk of non-functional overreaching, illness, and injury [10]. However, evidence detailing the training process and its impact on physical fitness in world-class para athletes is scarce, particularly longitudinal monitoring of how strength, power, and body composition adapt to periodized training in world-class para throwers. Crucially, no studies have quantified whether these adaptations differ between seated (e.g., F54) and standing (e.g., F42) throwers, despite their distinct biomechanical demands. Therefore, the aim of this study was to assess world-class shot put para athletes’ changes in physical fitness during six months of training, providing empirical data for coaches to evaluate training effectiveness in this population.

## 2. Materials and Methods

### 2.1. Experimental Design and Approach to the Problem

This study followed a longitudinal case report design, tracking two world-class throwing para athletes (shot-putters) throughout a 24-week training season using a longitudinal case report design. Physical fitness variables were assessed once per month over the training season; at the end of each training mesocycle, athletes were tested (with tests conducted always in the same sequence) on the last week of each month on the same day (Monday), starting at the beginning of the training season (February) with the final assessment taking place in July (a total of 6 time points). Athletes were instructed to avoid strenuous or unaccustomed exercise 48 h prior to testing. All tests were completed in a single day between 10:00 and 14:00. All testing sessions were supervised by an experienced strength and conditioning coach with expertise in training athletes with physical impairments. Athletes were verbally encouraged to exert maximum effort throughout the tests that were highly challenging (i.e., throwing, power–load relationship of the arm extensor muscles, and 3RM bench press [3RMBP]). To be eligible for analysis, subjects were required to attend over 90% of their training sessions and 100% of the testing sessions. The participants in this study were already familiar with the exercises and tests involved, having prior experience using them either in resistance training or throwing sessions.

### 2.2. Participants

One male (age: 34.8 years; body mass: 96.9 kg; height: 1.79 m; throwing experience: 2 years; competition shot put weight: 6 kg), and one female (age: 45.3 years; body mass: 60.1 kg; height: 1.64 m; throwing experience: 5 years; competition shot put weight: 3 kg) participated in the study. Inclusion criteria were as follows: (1) an active member of the Chilean Para Athletics national team; (2) ≥2 years of competitive experience in any Para Athletics throwing event; (3) regular training attendance (>90% of sessions); and (4) no planned interruptions during the study period. Exclusion criteria included the following: (1) acute injuries preventing normal training; (2) use of performance-enhancing substances; and (3) changes in classification or assistive equipment during the study. Both athletes were free of injuries or medical conditions limiting training participation, and confirmed they were not taking any ergogenic supplements beyond basic nutritional support (vitamins, protein powder, and creatine). The male athlete (F42 sport class for athletes with lower limb impairments competing in standing throwing events) had 2 years of throwing experience and won a silver medal. The female athlete (F54 sport class for athletes competing in a seated position) had 5 years of experience and ranked 4th at the Santiago 2023 Para Pan-American Games. Both para athletes were ranked first in their respective sport classes at the national level and were also competing at the international level. Athletes were healthy during the study and had no training injuries or other physical conditions that limited their involvement in regular shot put para athlete training. Written informed consent was obtained from participants before testing.

### 2.3. Training

The training program followed by the athletes (Table 1) was structured according to the principles of periodization [16]. Specifically, the entire training program was separated into two long-term periods: the general preparation phase and the specific preparation phase. The focus of the first training phase was to enhance athletes’ training ability, involving high-volume activity, aimed at basic adaptations. The second training phase aimed to cover sport-specific adaptations mainly by increasing the intensity of all training programs, including throwing and resistance training. The last month of training included an overload training period and the tapering phase. The overload training period aimed to increase volume and intensity in an attempt to stimulate adaptations [24], while, on the contrary, the tapering phase aimed to reduce training volume and fatigue as well as prepare athletes for the competition.

### 2.4. Throwing Performance

Throwing performance was assessed through a simulated competition protocol. After a 5–8 min warm-up (dynamic stretching for the upper and lower body; a series of 2–3 progressive practice throws with submaximal effort), athletes performed 6 maximum attempts aiming to throw the shot put as far as possible. Rest intervals between attempts followed World Para Athletics competition rules according to each athlete’s classification: 1 min for the F54 female athlete and 5 min for the F42 male athlete [25].

### 2.5. Power–Load Relationship of the Arm Extensor Muscles

Athletes performed a seated medicine ball throwing test [22]. Four medicine balls with weights ranging between 2 and 5 kg were used. Seated on the floor with their back positioned against the wall and knees straight, the athletes performed a maximum throw from the chest. Athletes performed maximal-effort throws using balls presented in a randomized sequence (generated via Research Randomizer, www.randomizer.org) to eliminate order effects. Athletes were instructed to throw each ball as far as possible under standardized conditions. The throw distance was measured to the nearest centimeter using chalk-marked landing points, with measurements taken from the front edge of the throwing line to the closest point of the ball’s impact. Two trials were made with each ball, with a one-minute rest between throws. The mean and maximum performances were used for analysis. The mean performance across all medicine ball throws was computed and presented as the combined seated ball throw [22].

### 2.6. Upper-Body Strength

The 3RMBP was used to monitor changes in upper-body strength, since the bench press is a common exercise used to measure upper-body strength in throwers [18,26,27]. Lombardi’s equation was used to estimate the one-repetition maximum (1RM) [28]. Upon the completion of a general warm-up session, an exercise-specific warm-up was completed, including 8 repetitions with an empty bar, followed by two sets of 5 repetitions, and then 3 repetitions, all at submaximal self-selected loads. Each subject had three attempts to achieve a 3RMBP load, with minimum increments of 2.5 kg between trials, and three minutes of rest between trials [29]. Two spotters were present for all maximal efforts.

### 2.7. Body Composition

Height (cm) was recorded to the nearest 0.1 cm using a stadiometer (SECA GmbH, Hamburg, Germany). Body mass was measured using a Seca scale (SECA GmbH, Hamburg, Germany), taken to the nearest 0.1 kg; BMI was calculated as weight/height^2^ (kg/m^2^). A Harpenden caliper (Baty International, Burgess Hill, UK) was used to measure biceps, triceps, subscapular, abdominal, suprailiac, and supraspinal skinfolds on the right side (dominant). Measurements were conducted following previously published procedures [30]. Consistent with prior expert recommendations encouraging raw anthropometric data [31], we reported both the sum of six skinfolds (mm) and the combined arm skinfold measurement (mm).

### 2.8. Statistical Analyses

Descriptive statistics [the mean, standard deviation (SD), and minimum–maximum] were calculated to summarize and provide an overview of the data distribution of all variables. The coefficient of variation (CV) was calculated as (standard deviation/mean) × 100, providing a normalized measure of variability relative to the mean performance. The percentage of change (%) was determined to examine changes over time.

## 3. Results

The para athletes trained for an average of 1.5 ± 0.39 h per day for 5–6 days per week. The results for the shot put throwing test are presented in Table 2. Both athletes increased their competitive throwing performance during the training period.

**Figure 1:**
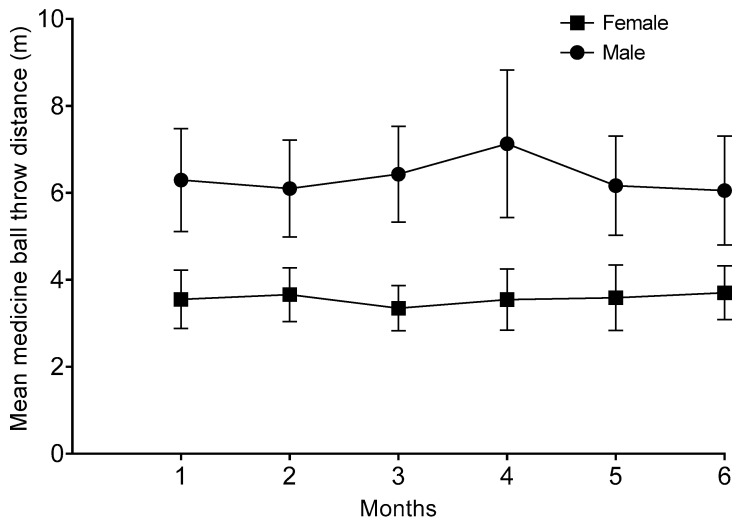
Mean performance across all seated medicine ball throws over 6 months.

Table 3 presents the results for the power–load relationship of the arm extensor muscles, both for the male and female athletes. The male athlete produced the highest medicine ball throw values during the 4th month, while the female athlete demonstrated progressive increases across the training period. Moreover, the average performances across all ball throws for both athletes are presented in Figure 1.

Table 4 presents the increases in 3RMBP for both athletes. The 1RM bench press (1RMBP) was subsequently estimated using Lombardi’s equation (1RM = weight × (repetitions)^0.10) [28].

Table 5 presents the results from the body composition analysis, where both athletes decreased their body sum from six skinfolds.

## 4. Discussion

The purpose of this study was to assess world-class shot put para athletes’ physical fitness changes during six months of training, providing empirical data for coaches to evaluate training effectiveness in this population. The findings of this monitoring study indicated that both male and female athletes experienced improvements in upper-body strength, power output, and competitive shot put throwing performance, with fluctuations across the training cycle. These results align with previous research, reinforcing the importance of periodized strength–power training for maximizing strength, power, and throwing performance [32,33]. In addition, both athletes finished the training cycle without injuries, which further reinforces the applicability of the current training program in shot put para athletes. Moreover, the observed variations in monthly performance also highlight the challenges of maintaining consistent progress in para athletes, possibly due to the unique barriers they face (e.g., pathophysiological and psychosocial factors) [34,35]. Therefore, 24 weeks of training improved physical fitness in world-class shot put para athletes.

Shot put throwing performance increased by 10.1% for the male and 1.6% for the female shot-putter. These increases in shot put throws are both within the range of performance changes in non-disabled athletes [22,36]. The male shot-putter experienced greater improvement during the 5th month of training, followed by a slight decline (~10 cm) in the final month of training. This small decrease in shot put performance, especially in para athletes, may be due to several reasons such as technical problems, accumulated fatigue, or even a sudden reduction in training load, which might have led to a loss of fitness [37,38]. By contrast, the female shot-putter had a lower response in shot put performance following the training program compared to the male para athlete. These differences could be partially attributed to the distinct throwing positions used (standing vs. seated). In addition, other factors like training age (5 vs. 2 years), sex-specific physiological profiles, and impairment characteristics may also contribute. This exploratory hypothesis warrants verification in larger samples. The male para athlete competed in a standing position with a knee impairment that limits flexion (reduced range of motion), while the female para athlete throws in a sitting position due to a spinal cord injury. We can hypothesize that the male athlete was able to activate a greater part of his muscle mass as well as achieve higher velocities during the throw, while the female para athlete was able to involve only the upper body musculature system and produce lower velocities during the throwing movement. Additionally, it is well documented that female athletes have lower lean mass, strength, and power compared to male athletes [39], and these differences are evident even when strength and power are expressed relative to body mass [40,41]. However, such differences between para athletes have not been investigated yet [42].

An upper-body muscle power assessment in both para athletes and healthy exercisers is a vital measurement for evaluating the throwing capacity [13]. In the current study, the seated medicine ball was used as an upper-body power measurement [22]. As was expected, the seated medicine ball throw test revealed load-dependent power adaptations. Interestingly, the male shot-putter achieved his highest throws with all medicine ball weights during the 4th month of training. Power adaptations varied by mesocycle: the male athlete showed peak medicine ball throws during the specific phase (Month 4), while the female demonstrated linear progress across all six mesocycles (Table 3), aligning with their distinct periodization needs. This finding may be related to the focus of training during the last 2 months of the preparation, which shifted more to the whole-body throwing capacity using multi-joint throwing exercises (backward and underhand shot put throws) [3]. Moreover, the subsequent decline may indicate overreaching or insufficient recovery, which is a risk previously noted in para athletes due to their higher susceptibility to illness and overtraining [43,44]. On the other hand, the female shot-putter experienced a continuous increase in seated medicine ball throwing from the beginning to the end of the training period in all medicine ball throw weights. The female shot-putter competed from a seated position, which is closer to the seated medicine ball throw. In addition, the upper body musculature system is involved in both shot put and medicine ball throwing. Therefore, for seated para athletes, the seated medicine ball throw test might be more representative of monitoring training-induced adaptations and the increases in competitive shot put throwing performance. Furthermore, the above results suggest the need for individualized load management, as female throwers often exhibit different strength–power development patterns compared to males [45,46].

In general, muscle strength is a significant factor for shot put performance both in male and female athletes [3,44]. In the current study, both para athletes improved their 3RMBP performance (male: 10.4%; female: 3.3%), reinforcing the critical role of upper-body strength in shot put success [19,47]. The male para athlete increased their whole-body mass (2.5%) alongside a reduction in Σ6SKF (9.8%), suggesting favorable lean mass accrual, consistent with resistance training adaptations [48,49]. Lean mass is a strong predictor of shot put performance in non-disabled athletes [50,51], which seems to be repeated in para athletes. In addition, there is a strong link between lean mass and strength [52], which underpins the fact that increases in muscle strength may accompany positive changes in lean mass and shot put performance. Conversely, the female athlete’s decreased body mass (2.5%) and Σ6SKF (11.7%) may reflect reductions in body fat and optimized power-to-mass ratio adjustments, which have been linked to improved throwing performance in female shot-putters [36,53,54]. These results suggest that coaches should regularly monitor body composition and upper-body muscle strength to assess the readiness of their athletes.

### Limitations and Future Directions

In light of this study’s aims to assess training effects and provide empirical benchmarks for elite para throwers, several limitations warrant discussion: while this study provides valuable insights into a specific population of throwing para athletes, the small sample size, although including world-class athletes in their categories, limits the external validity of the results and their utility as population benchmarks. These results cannot be generalized to other populations without further research. These data should serve as a foundation for future large-scale normative studies. Additionally, tests were supervised by the athlete’s training team to ensure ecological validity, which may introduce observer bias. While sRPE data were collected, this study did not quantify internal training load responses (e.g., heart rate or perceived exertion) or recovery markers (e.g., HRV, CK, and cortisol level), focusing instead on external load and performance adaptations. Future studies should integrate both dimensions. However, recruiting para throwers and interfering in their daily training program is a challenging procedure. While we tracked training and testing consistency (time of day, monthly intervals), we did not assess menstrual cycle phase in the female athlete, despite evidence that hormonal fluctuations may differentially impact strength and anaerobic performance [55]. Future studies should aim to (1) incorporate this variable when monitoring female para athletes and (2) include larger and more diverse samples to improve the generalizability of the findings, including fatigue biomarkers (e.g., cortisol, testosterone) to assess the influence of training loads and extend longitudinal tracking across multiple seasons to assess chronic adaptations.

## 5. Conclusions

World-class shot put para athletes can achieve significant strength and power gains through structured periodized training (male: +10.1% in throws and +10.4% in 3RMBP; female: +1.6% in throws and +3.3% in 3RMBP), with performance varying between individuals. While primarily assessing training adaptations, coaches should prioritize individualized periodization, frequent power assessments (particularly seated medicine ball throwing for seated athletes, as our data suggest its reliability as a performance proxy), and careful fatigue management to maximize competitive outcomes. The data offered in this study provide empirical insights for coaches to optimize their own training programs and maximize throwers’ performance. Additionally, monitoring physical fitness and the periodization of training loads during year-round training provides information on the effectiveness of the training programs followed by athletes, helping to reduce injury risk factors and increase para athletes’ performance.

## Figures and Tables

**Table 1:** Characteristics of the 6-month training program.

	General Phase	Specific Phase	Overload Phase	Tapering Phase
Duration	3 months (3 mesocycles of 4 weeks)	2 months (2 mesocycles of 4 weeks)	2 weeks	2 weeks
Aim	Developing muscle hypertrophy, strength, and general athleticism	Developing sport-specific power and technique	Short intensification phase with increased volume and intensity to stimulate adaptation	Full recovery protocols (active recovery, mobility work, training volume <50%, peak performance)
Throws	2 technical sessions (shot put drills)	3–4 throwing sessions (heavy and competition-weight implements)	4–5 throwing sessions/week	3–4 high-quality throwing sessions (competition simulation)
Resistance training sessions	4–5 resistance training sessions (emphasis on compound lifts, e.g., military press and bench press)	3–4 resistance sessions (higher intensity but lower volume; addition of Olympic derivative lifts)	Near 1-RM attempts in basic multi-joint lifts	2–3 resistance sessions (maintenance loads)
Power/plyometric sessions	2–3 explosive/plyometric sessions (e.g., medicine ball throws and jumps)	2 plyometric/sprint sessions (focused on horizontal and vertical power)	2 plyometric/sprint sessions (focused on horizontal and vertical power)	1–2 plyometric/sprint sessions
Aerobic conditioning sessions	1–2 aerobic conditioning sessions (low-intensity running or circuits)			

**Table 2:** Shot put throwing test.

Month	Male					Female				
	Mean	SD	Max	CV	Δ%	Mean	SD	Max	CV	Δ%
1	9.51	0.36	9.84	3.7	-	6.76	0.10	6.88	1.0	—
2	9.29	0.27	9.58	2.9	−2.64	6.48	0.18	6.76	3.0	−1.74
3	10.34	0.22	10.56	2.1	10.23 *	6.43	0.13	6.63	2.0	−1.92
4	10.49	0.13	10.69	1.2	1.23 *	6.78	0.05	6.85	1.0	3.32 *
5	10.46	0.34	10.93	3.3	−0.29	6.67	0.12	6.84	2.0	−1.62
6	10.41	0.33	10.83	3.2	−0.91	6.83	0.13	6.99	2.0	2.19 *

Note: All distance measurements are in meters. SD = standard deviation; Max = best trial in the test; CV = coefficient of variation (%); Δ% = percentage of change compared to the previous month; * Indicates that the percentage change exceeds the coefficient of variation, representing a meaningful change beyond measurement errors.

**Table 3:** Medicine ball throw test for the male athlete.

Weight	Month	Mean (m)	Max (m)	SD (m)	CV (%)	Δ%
2 kg	1	7.53/4.40	7.76/4.40	0.33/0.01	4.42/0.16	—
	2	7.44/4.45	7.46/4.47	0.04/0.04	0.48/0.80	−3.87/1.59 *
	3	7.72/3.92	7.72/3.97	0.01/0.08	0.09/1.99	3.49 */−11.19 *
	4	8.07/4.24	8.12/4.43	0.07/0.28	0.88/6.51	5.18 */11.59 *
	5	7.57/4.45	7.61/4.52	0.06/0.11	0.75/2.39	−6.28 */2.03
	6	7.65/4.51	7.70/4.52	0.05/0.02	0.65/0.47	1.06 */0
3 kg	1	6.65/3.62	6.70/3.66	0.08/0.06	1.17/1.56	—
	2	6.47/3.79	6.52/3.81	0.08/0.03	1.20/0.75	−2.69 */4.1 *
	3	6.67/3.47	6.88/3.53	0.30/0.09	4.56/2.65	5.52 */−7.35 *
	4	7.04/3.64	7.09/3.75	0.07/0.16	1.01/4.27	−8.74 */6.23 *
	5	6.32/3.75	6.47/3.81	0.22/0.08	3.47/2.26	−28.03 */1.60
	6	6.28/3.72	6.30/3.76	0.03/0.06	0.45/1.71	−2.63/−1.31
4 kg	1	5.54/3.33	5.61/3.36	0.11/0.04	1.92/1.27	—
	2	5.41/3.07	5.46/3.23	0.07/0.23	1.31/7.61	−2.67 */−3.87 *
	3	5.77/3.06	5.91/3.13	0.21/0.11	3.56/3.47	8.24 */−3.10
	4	5.90/3.08	5.94/3.19	0.06/0.16	1.08/5.29	0.51/1.92
	5	5.40/3.12	5.59/3.25	0.27/0.19	4.98/6.13	−5.89 */1.88
	6	5.34/3.43	5.37/3.49	0.05/0.08	0.93/2.47	−3.94/7.38 *
5 kg	1	4.96/2.73	5.11/2.78	0.21/0.07	4.28/2.59	—
	2	4.88/3.04	4.96/3.12	0.11/0.11	2.32/3.72	−2.94/12.23 *
	3	5.15/2.73	5.21/2.76	0.08/0.04	1.65/1.55	5.04 */−11.54 *
	4	5.26/2.77	5.47/2.81	0.30/0.06	5.65/2.30	4.99 */1.81 *
	5	4.92/2.75	4.99/2.77	0.10/0.04	2.01/1.29	−8.78 */−1.42
	6	4.73/2.82	4.84/3.04	0.16/0.31	3.29/11.03	−3.01 */9.75 *

Values represent male/female measurements (presented as male value/female value) for all parameters. Δ% = percentage of change compared to the previous month; * indicates that the percentage change exceeds the coefficient of variation, representing a meaningful change beyond measurement errors.

**Table 4:** Three-repetition maximum (3RMBP) and estimated one-repetition maximum (1RM) for male and female athletes over six months.

Month	Male			Female		
	3RM	E1RM	Δ%	3RM	E1RM	Δ%
1	125.0	139.5	—	60.0	67.0	—
2	130.0	145.1	+4.0%	55.0	61.4	−9.1%
3	135.0	150.7	+3.8%	60.0	67.0	+10.0%
4	135.0	150.7	0%	62.0	69.2	+3.3%
5	130.0	145.1	−3.7%	62.0	69.2	0%
6	138.0	154.0	+6.1%	62.0	69.2	0%

E1RM = estimated one-repetition maximum calculated using Lombardi’s formula; Δ% = percentage change compared to the previous month.

**Table 5:** Body composition and upper-body strength.

	Before *		After *		Δ Male	Δ Female
	Male	Female	Male	Female		
Body mass (kg)	96.9	60.1	99.3	58.6	+2.4 (+2.5%)	−1.5 (−2.5%)
Body mass index (kg∙m^−2^)	30.0	22.3	31.0	21.8	+1.0 (+3.3%)	−0.5 (−2.2%)
Σ6SKF (mm)	127.5	111.0	115.0	98.0	−12.5 (−9.8%)	−13.0 (−11.7%)
Σ_arm_SKF (mm)	22.5	18.0	23.0	20.0	+0.5 (+2.2%)	+2.0 (+11.1%)

Δ = absolute change (percentage change in parentheses); Σ6SKF = sum of six skinfolds (biceps, triceps, subscapular, abdominal, suprailiac, and supraspinal folds); Σ_arm_SKF = combined arm skinfolds (biceps and triceps); * = before and after 6 months of training.

## Data Availability

All data generated or analyzed during this study are included in the article as Tables or Figures. Additional requirements can be directed to the corresponding author upon reasonable request, subject to privacy and ethical considerations.

## Abbreviations

The following abbreviations are used in this manuscript:

1RM	One-repetition maximum
3RMBP	Three-repetition maximum bench press
BMI	Body mass index
CV	Coefficient of variation
E1RM	Estimated one-repetition maximum
SD	Standard deviation
Σ6SKF	Sum of six skinfolds
ΣarmSKF	Sum of arm skinfolds
sRPE	Session rate of perceived exertion
Δ%	Percentage change

## References

[1] Judge L.W. (2007). Developing Speed Strength: In-Season Training Program for the Collegiate Thrower. Strength Cond. J..

[2] Hu B. (2023). Effects of variable exercise on strength training in throwing athletes. Rev. Bras. Med. Esporte.

[3] Anousaki E., Zaras N., Stasinaki A.-N., Panidi I., Terzis G., Karampatsos G. (2021). Effects of a 25-Week Periodized Training Macrocycle on Muscle Strength, Power, Muscle Architecture, and Performance in Well-Trained Track and Field Throwers. J. Strength Cond. Res..

[4] Simon S., Richards P. (2022). Individualising Coaching in Olympic and Paralympic Worlds: An Applied Perspective. Int. Sport. Coach. J..

[5] Hartmann H., Wirth K., Keiner M., Mickel C., Sander A., Szilvas E. (2015). Short-term Periodization Models: Effects on Strength and Speed-strength Performance. Sports Med..

[6] MacLachlan M., Banes D., Bell D., Borg J., Donnelly B., Fembek M., Ghosh R., Gowran R.J., Hannay E., Hiscock D. (2018). Assistive technology policy: A position paper from the first global research, innovation, and education on assistive technology (GREAT) summit. Disabil. Rehabil. Assist. Technol..

[7] Oggero G., Puli L., Smith E.M., Khasnabis C. (2021). Participation and Achievement in the Summer Paralympic Games: The Influence of Income, Sex, and Assistive Technology. Sustainability.

[8] Caplin A., Chen F.S., Beauchamp M.R., Puterman E. (2021). The effects of exercise intensity on the cortisol response to a subsequent acute psychosocial stressor. Psychoneuroendocrinology.

[9] Fox J.L., Stanton R., Sargent C., Wintour S.A., Scanlan A.T. (2018). The Association Between Training Load and Performance in Team Sports: A Systematic Review. Sports Med..

[10] Halson S.L. (2014). Monitoring Training Load to Understand Fatigue in Athletes. Sports Med..

[11] McGuigan H., Hassmén P., Rosic N., Stevens C.J. (2020). Training monitoring methods used in the field by coaches and practitioners: A systematic review. Int. J. Sports Sci. Coach..

[12] Wing C. (2018). Monitoring Athlete Load: Data Collection Methods and Practical Recommendations. Strength Cond. J..

[13] Zaras N., Spengos K., Methenitis S., Papadopoulos C., Karampatsos G., Georgiadis G., Stasinaki A., Manta P., Terzis G. (2013). Effects of Strength vs. Ballistic-Power Training on Throwing Performance. J. Sports Sci. Med..

[14] Kyriazis T., Methenitis S., Zaras N., Stasinaki A.N., Karampatsos G., Georgiadis G., Terzis G. (2022). Effects of Complex vs. Compound Training on Competitive Throwing Performance. J. Strength Cond. Res..

[15] Martínez-García D., Chirosa Ríos L., Rodriguez-Perea A., Ulloa-Díaz D., Jerez-Mayorga D., Chirosa Ríos I. (2021). Strength training for throwing velocity enhancement in overhead throw: A systematic review and meta-analysis. Int. J. Sports Sci. Coach..

[16] Stone M.H., Hornsby W.G., Haff G.G., Fry A.C., Suarez D.G., Liu J., Gonzalez-Rave J.M., Pierce K.C. (2021). Periodization and Block Periodization in Sports: Emphasis on Strength-Power Training-A Provocative and Challenging Narrative. J. Strength Cond. Res..

[17] Garcia-Carrillo E., Silva B., Zaras N., Ramirez-Campillo R. (2025). Training practices of male and female para athletics throwers: From developmental to world-class levels. Retos.

[18] Ojanen T., Rauhala T., Häkkinen K. (2007). Strength and power profiles of the lower and upper extremities in master throwers at different ages. J. Strength Cond. Res..

[19] Terzis G., Kyriazis T., Karampatsos G., Georgiadis G. (2012). Muscle strength, body composition, and performance of an elite shot-putter. Int. J. Sports Physiol. Perform..

[20] Garcia-Carrillo E., Silva B., Zaras N., Azocar-Gallardo J., Yáñez-Sepúlveda R., Ramirez-Campillo R. (2024). Prevalence of sports injuries in Para Athletics throwers-a retrospective cohort study. Adv. Rehabil..

[21] Sánchez-Medina L., González-Badillo J.J., Pérez C.E., Pallarés J.G. (2014). Velocity- and power-load relationships of the bench pull vs. bench press exercises. Int. J. Sports Med..

[22] Zaras N., Stasinaki A.-N., Arnaoutis G., Terzis G. (2016). Predicting throwing performance with field tests. New Stud. Athl..

[23] Bourdon P.C., Cardinale M., Murray A., Gastin P., Kellmann M., Varley M.C., Gabbett T.J., Coutts A.J., Burgess D.J., Gregson W. (2017). Monitoring Athlete Training Loads: Consensus Statement. Int. J. Sports Physiol. Perform..

[24] Thomas L., Mujika I., Busso T. (2009). Computer simulations assessing the potential performance benefit of a final increase in training during pre-event taper. J. Strength Cond. Res..

[25] International Paralympic Committee (2024). World Para Athletics Rules and Regulations 2024.

[26] Terzis G., Karampatsos G., Georgiadis G. (2007). Neuromuscular control and performance in shot-put athletes. J. Sports Med. Phys. Fit..

[27] Caughey R.M., Thomas C. (2022). Variables Associated with High School Shot Put Performance. Int. J. Exerc. Sci..

[28] Ribeiro Neto F., Guanais P., Dornelas E., Coutinho A.C.B., Costa R.R.G. (2017). Validity of one-repetition maximum predictive equations in men with spinal cord injury. Spinal Cord.

[29] Weakley J.J.S., Till K., Darrall-Jones J., Roe G.A.B., Phibbs P.J., Read D.B., Jones B.L. (2017). The Influence of Resistance Training Experience on the Between-Day Reliability of Commonly Used Strength Measures in Male Youth Athletes. J. Strength Cond. Res..

[30] Garcia-Carrillo E., Yáñez-Sepúlveda R., Cortés-Roco G., Ramirez-Campillo R., Izquierdo M. (2023). Anthropometric characteristics, handgrip strength, and upper limb asymmetries in highly trained Chilean shot put para-athletes. Int. J. Morphol..

[31] Broad E. (2014). Sports Nutrition for Paralympic Athletes.

[32] DeWeese B.H., Hornsby G., Stone M., Stone M.H. (2015). The training process: Planning for strength–power training in track and field. Part 1: Theoretical aspects. J. Sport. Health Sci..

[33] Suchomel T.J., Nimphius S., Bellon C.R., Stone M.H. (2018). The Importance of Muscular Strength: Training Considerations. Sports Med..

[34] Wilson N.C., Khoo S. (2013). Benefits and barriers to sports participation for athletes with disabilities: The case of Malaysia. Disabil. Soc..

[35] Liu J., Yu H., Cheung W.C., Bleakney A., Jan Y.-K. (2025). A systematic review of pathophysiological and psychosocial measures in adaptive sports and their implications for coaching practice. Heliyon.

[36] Anousaki E., Stasinaki A.N., Zaras N., Terzis G., Methenitis S., Arnaoutis G., Karampatsos G. (2018). Rate of force development, lean body mass and throwing performance in female shot-put athletes. J. Phys. Educ. Sport..

[37] Mujika I., Padilla S. (2003). Scientific bases for precompetition tapering strategies. Med. Sci. Sports Exerc..

[38] Garcia-Carrillo E., Ramirez-Campillo R. (2020). Peaking for the World Para Athletics Championships: Case study of a World Champion female Paralympic shot putter. J. Hum. Sport. Exerc..

[39] Bishop P., Cureton K., Collins M. (1987). Sex difference in muscular strength in equally-trained men and women. Ergonomics.

[40] Harbili E. (2012). A gender-based kinematic and kinetic analysis of the snatch lift in elite weightlifters in 69-kg category. J. Sports Sci. Med..

[41] Bartolomei S., Grillone G., Di Michele R., Cortesi M. (2021). A Comparison between Male and Female Athletes in Relative Strength and Power Performances. J. Funct. Morphol. Kinesiol..

[42] O’Connor S.R., Fagher K., Williamson S., Pluim B.M., Ardern C.L., Janse van Rensburg D.C., Heron N. (2022). Assessment of muscle strength in para-athletes: A systematic review of observational studies. Sports Med. Health Sci..

[43] Schwellnus M., Soligard T., Alonso J.-M., Bahr R., Clarsen B., Dijkstra H.P., Gabbett T.J., Gleeson M., Hägglund M., Hutchinson M.R. (2016). How much is too much? (Part 2) International Olympic Committee consensus statement on load in sport and risk of illness. Br. J. Sports Med..

[44] Keaney L.C., Kilding A.E., Merien F., Dulson D.K. (2019). Keeping Athletes Healthy at the 2020 Tokyo Summer Games: Considerations and Illness Prevention Strategies. Front. Physiol..

[45] Nuzzo J.L. (2023). Narrative Review of Sex Differences in Muscle Strength, Endurance, Activation, Size, Fiber Type, and Strength Training Participation Rates, Preferences, Motivations, Injuries, and Neuromuscular Adaptations. J. Strength Cond. Res..

[46] Augustsson J., Gunhamn T., Andersson H. (2024). An Assessment of the Ratio between Upper Body Push and Pull Strength in Female and Male Elite Swedish Track and Field Throwers. Sports.

[47] Soares D., Lourenço J., Silva A.F., Flôres F. (2023). Influence of Maximal Strength on Bench Press and Trunk Rotation in Adapted Shot-put: A Pilot Investigation. Exerc. Sci..

[48] Helms E.R., Spence A.-J., Sousa C., Kreiger J., Taylor S., Oranchuk D.J., Dieter B.P., Watkins C.M. (2023). Effect of Small and Large Energy Surpluses on Strength, Muscle, and Skinfold Thickness in Resistance-Trained Individuals: A Parallel Groups Design. Sports Med.-Open.

[49] Androulakis Korakakis P., Wolf M., Coleman M., Burke R., Piñero A., Nippard J., Schoenfeld B.J. (2024). Optimizing Resistance Training Technique to Maximize Muscle Hypertrophy: A Narrative Review. J. Funct. Morphol. Kinesiol..

[50] Zaras N., Stasinaki A.-N., Terzis G. (2021). Biological Determinants of Track and Field Throwing Performance. J. Funct. Morphol. Kinesiol..

[51] Terzis G., Georgiadis G., Vassiliadou E., Manta P. (2003). Relationship between shot put performance and triceps brachii fiber type composition and power production. Eur. J. Appl. Physiol..

[52] Tromaras K., Zaras N., Stasinaki A.-N., Mpampoulis T., Terzis G. (2024). Lean Body Mass, Muscle Architecture and Powerlifting Performance during Preseason and in Competition. J. Funct. Morphol. Kinesiol..

[53] Landolsi M., Bouhlel E., Zarrouk F., Lacouture P., Tabka Z. (2014). The relationships between leg peak power and shot-put performance in national-level athletes. Isokinet. Exerc. Sci..

[54] Kyriazis T., Terzis G., Karampatsos G., Kavouras S., Georgiadis G. (2010). Body Composition and Performance in Shot Put Athletes at Preseason and at Competition. Int. J. Sports Physiol. Perform..

[55] Carmichael M.A., Thomson R.L., Moran L.J., Wycherley T.P. (2021). The Impact of Menstrual Cycle Phase on Athletes’ Performance: A Narrative Review. Int. J. Environ. Res. Public Health.

